# Enhanced Room Temperature NO_2_ Sensing Performance of RGO Nanosheets by Building RGO/SnO_2_ Nanocomposite System

**DOI:** 10.3390/s19214650

**Published:** 2019-10-26

**Authors:** Hongfei Du, Guangzhong Xie, Qiuping Zhang

**Affiliations:** 1 School of Optoelectronic Science and Engineering, State Key Laboratory of Electronic Thin Films and Integrated Devices, University of Electronic Science and Technology of China (UESTC), Chengdu 610054, China; hfdu@uestc.edu.cn (H.D.); gzxie@uestc.edu.cn (G.X.); 2 School of Mathematical Sciences, University of Electronic Sciences and Technology of China (UESTC), Chengdu 611731, China; 3 Key Laboratory of Information Materials of Sichuan Province, School of Electrical and Information Engineering, Southwest University for Nationalities, Chengdu 610041, China

**Keywords:** nitrogen dioxide, RGO/SnO_2_ composite, airbrush, anneal, room temperature

## Abstract

RGO/SnO${}_{2}$ nanocomposites were prepared by a simple blending method and then airbrushed on interdigitated electrodes to obtain the corresponding gas sensors. The characterizations of SEM, TEM, Raman, XRD and FTIR were used to characterize the microstructures, morphologies and surface chemical compositions of the nanocomposites, indicating that the two materials coexist in the composite films and the concentration of surface defects is affected by the amount of SnO${}_{2}$ nanoparticles. It is also found that the room temperature sensing performance of RGO to NO${}_{2}$ can be improved by introducing appropriate amount of SnO${}_{2}$ nanoparticles. The enhanced NO${}_{2}$ sensing properties are attributed to the rough surface structure and increased surface area and surface defects of the nanocomposite films. Since further reduction of RGO, heat treating the sensing films resulted in a decrease in the response and recovery times of the sensors. Furthermore, the sensor annealed at 200 ${}^{\circ }$C exhibited a small baseline drift, wide detection range, good linearity, high stability and better selectivity.

## 1. Introduction

Nitrogen dioxide (NO${}_{2}$), a well-known toxic and harmful gas with a pungent odor, is a prominent air pollutant and one of the main causes of acid rain. It mainly comes from automobile exhaust and factory emissions, which is usually harmful to human beings, animals and plants. Approximately 10–20 ppm NO${}_{2}$ can cause mild irritation of the nose and throat, and concentrations above 50 ppm are considered dangerous to man for short exposures [1,2]. Therefore, the detection of low concentrations of NO${}_{2}$ with high sensitivity at room temperature is highly desirable.

Metal oxide gas sensors have been extensively studied and commercially applied. As an n-type semiconducting metal oxide material [3], tin oxide (SnO${}_{2}$) is sensitive to many reducing and oxidizing gases (especially NO${}_{2}$), and is widely used for its low cost, no toxicity and high chemical stability. In general, the small size nanoparticles possess a large surface area and, thus, can adsorb gas molecules easily and improve sensing properties. However, SnO${}_{2}$ nanoparticles tend to aggregate together, thereby hindering the diffusion of gas molecules on the surface of sensitive films [4]. Meanwhile, the cross sensitivity with other gases, high operating temperature and long recovery time limit its application in NO${}_{2}$ detection [4,5,6,7].

In recent years, graphene and its derivatives (such as graphene oxide (GO) and reduced graphene oxide (RGO) etc.) have shown great potential in chemical- and bio-sensors because of their low cost, high specific surface area, low electrical noise and high carrier mobility at room temperature [4,5]. To further improve the performances of SnO${}_{2}$ gas sensors, graphene and RGO are usually composited with SnO${}_{2}$ nanoparticles, and its superiority has been demonstrated by some studies [4,5,6,8,9,10].

The metal oxide/graphene composite materials were prepared by many methods such as the gas–liquid interfacial solvothermal method [5], the hydrothermal method [4,9,10], the sol–gel method [6,11], and the one pot synthesis method [8] etc. The chemical reagents used in the synthesis are expensive, some of them are toxic, and the preparation process is complex. Large-scale industrial production requires simple preparation methods, but there are few studies in this area.

In this paper, different amount of SnO${}_{2}$ nanopowders were simply blended with diluted RGO aqueous dispersion (0.0215 wt%), and the pure RGO and RGO/SnO${}_{2}$ composites were deposited on interdigitated electrodes (IDEs) with airbrush technology. Their microstructures, surface morphologies and surface chemical compositions, as well as NO${}_{2}$ sensing properties, were investigated.

## 2. Experimental

### 2.1. Materials Synthesis and Sensor Fabrication

**Figure 1:**
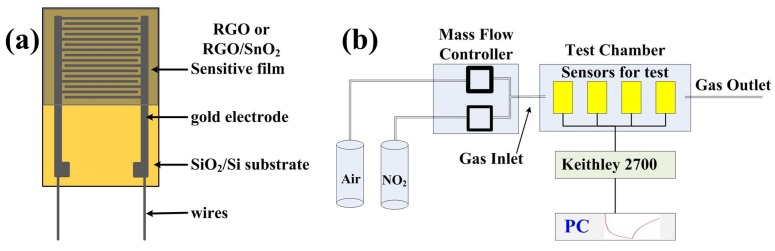
Schematic illustration of (**a**) the as-fabricated gas sensors and (**b**) measurement setup.

RGO aqueous dispersion (0.43 wt%, RGO: 96.41% C, 3.59% O, 1–10 layers) was purchased from Chengdu Organic Chemicals Co. Ltd., Chinese Academy of Sciences (Chengdu, China). SnO${}_{2}$ nanopowder (purity 99.5%) was purchased from Chengdu Kelong Chemical Reagent Factory (Chengdu, China). In a typical synthesis, 2 mL RGO aqueous dispersion (0.43 wt%) was diluted to 1/20 by using 38 mL deionized water, and then sonicated for 20 min to obtain a uniform dispersion (0.0215 wt%). After that, the different amounts (0, 20, 40 and 80 mg) of SnO${}_{2}$ nanopowders were added into 10 mL diluted RGO dispersion (0.0215 wt%) and ultrasonicated for 20 min to obtain the composite solutions that the weight percentages of RGO were 100%, 9.7%, 5.1% and 2.6%. 0.5 mL of RGO, 9.7%RGO/SnO${}_{2}$, 5.1%RGO/SnO${}_{2}$ and 2.6%RGO/SnO${}_{2}$ aqueous dispersions were respectively airbrushed onto interdigital electrodes (IDEs), and finally dried in a vacuum oven at 70 ${}^{\circ }$C for an hour to fabricate NO${}_{2}$ sensors. Figure 1a depicts a schematic of the as-fabricated gas sensors. The IDE has a size of 7mm×11mm, and 20-nm-thick titanium is thermally evaporated on SiO${}_{2}$(300 nm)/Si, and then 100-nm-thick gold is evaporated thereon. The finger width and gap width of the IDE are both 0.05 mm.

### 2.2. Material Characterization

The morphologies of the as-prepared sensitive films were observed by a Hitachi S4800 field emission scanning electron microscope (FESEM, Tokyo, Japan) operated at an acceleration voltage of 20 kV. The microstructures were characterized on a FEI Tecnai G2 F20 transmission electron microscope (TEM, Hillsboro, OR, USA) equipped with energy dispersive X-ray (EDX) spectrometry at an acceleration voltage of 200 kV. The phase composition was identified by grazing incidence X-ray diffraction (XRD) technique on a Panalytical X’Pert Pro MPD diffractometer (Eindhoven, Holland) with Cu K${}_{\alpha }$ radiation (λ=0.15418 nm). Raman spectra were recorded using a Renishaw RM2000 Raman microscope spectrometer (Gloucestershire, UK) with an excitation wavelength of 514.5 nm at laser power of 10 mW. The surface chemical states were investigated by Fourier transform infrared spectra (FTIR) on a Bruker Vertex70 FTIR spectrometer (Ettlingen, Germany) in the frequency range of 600–4000 cm${}^{-1}$ with a resolution of 2 cm${}^{-1}$.

### 2.3. Test Instrument and Measurement Procedure

The testing apparatus for gas sensing is shown in Figure 1b. Gas concentration was controlled by a mass flow controller (MFC300, Suzhou Aitoly Electronic Equipment Co., Ltd., Suzhou, China). Dry air was used as the carrier, dilution, and purge gas. All experimental results were obtained at room temperature (298.15 K). During NO${}_{2}$ sensing tests, the sensors were fixed into a little sealed metal chamber and then purged by dry air to ensure a negligible effect of ambient humidity on the sensors’ performance. The real-time resistance (denoted as *R*) of the sensors were obtained by Keithley 2700 multimeter/Data Acquisition System at a constant flow rate of 100 mL/min, when NO${}_{2}$ concentration changed from 10 to 50 ppm.

In this paper, sensing response is defined as

(1) $$\left|\Delta R\right|/R_{0}=\left|R_{g}-R_{0}\right|/R_{0}$$

$R_{g}$ and $R_{0}$ represent respectively the resistance when the sensors are exposed to target gas and dry air.

When the resistance of sensor changes from the initial value to 90% of the total resistance change during the adsorption process, the corresponding time is response time. While the recovery time is the time that the resistance of sensor recovers 90% of the total resistance change during the desorption process [10].

## 3. Results and Discussion

### 3.1. Characterizations

**Figure 2:**
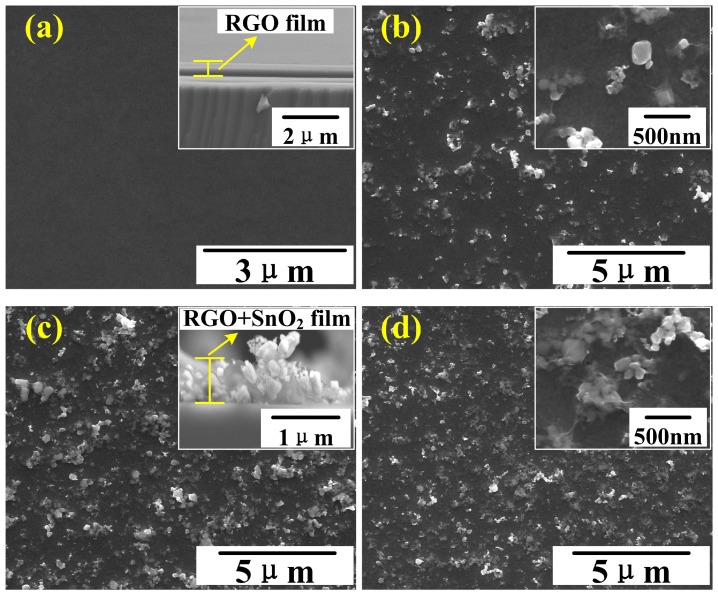
(**a**–**d**) SEM images of RGO, 9.7%RGO/SnO${}_{2}$, 5.1%RGO/SnO${}_{2}$ and 2.6%RGO/SnO${}_{2}$ film. The inset figures in (**a**,**c**) are cross-section images. The inset figures in (**b**,**d**) are magnification images.

The SEM images in Figure 2a–d present the surface morphologies of RGO film and RGO/SnO${}_{2}$ nanocomposite films prepared by air-brush method. The SnO${}_{2}$ nanoparticles are about 20–100 nm in size, which are encapsulated by single or multilayer RGO sheets.

The pure RGO film shown in Figure 2a exhibits a flat and featureless surface morphology. No gap between the two sides is observed, when the RGO sheets were deposited directly on IDEs (inset in Figure 2a).

After introducing SnO${}_{2}$ nanoparticles, it can be seen from Figure 2b–d that the surfaces of RGO/SnO${}_{2}$ nanocomposite films become uneven and rough. Further observation indicates that the SnO${}_{2}$ nanoparticles in the films become more and more dense with increasing the SnO${}_{2}$ content, and have a more even distribution. It is noteworthy that the introduction of SnO${}_{2}$ particles prevents not only the agglomeration of SnO${}_{2}$ particles [4] but also the stack of RGO sheets. These improvements increased the pores in films, as shown in the inset of Figure 2c.

**Figure 3:**
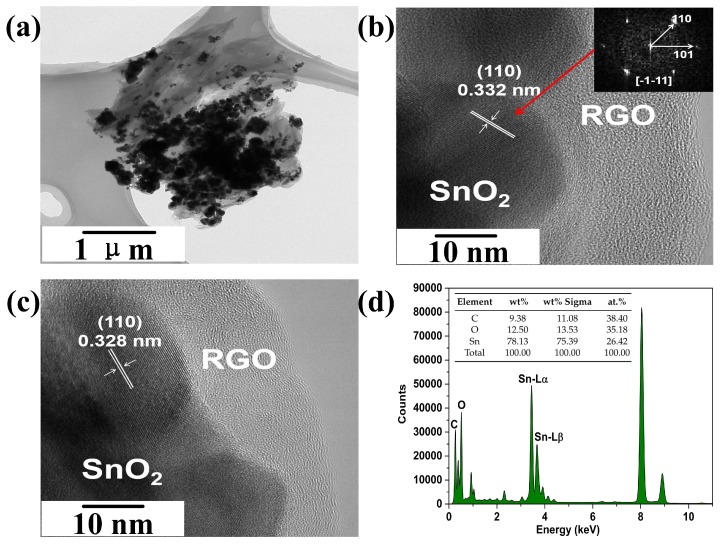
(**a**) TEM image, (**b**,**c**) HRTEM images and (**d**) EDX spectra of 5.1%RGO/SnO${}_{2}$ nanocomposites. Inset in (**b**) presents the corresponding processed reduced FFT pattern of the HRTEM image.

Figure 3a–c give the TEM and high-resolution TEM (HRTEM) images of 5.1%RGO/SnO${}_{2}$ nanocomposites, where the SnO${}_{2}$ nanoparticles are decorated on the multilayer RGO nanosheets, indicative of the successful formation of RGO/SnO${}_{2}$ nanocomposites. The lattice fringe spacings of 0.332 and 0.328 nm correspond to those of the (110) plane of SnO${}_{2}$ nanocrystal. To analyze the elementary composition of the nanocomposites, the EDX spectra were recorded, the corresponding results are shown in Figure 3d. It can be seen that the RGO/SnO${}_{2}$ nanocomposites are composed of C, O and Sn elements. In addition to O element associated with RGO, the atomic ratio of surplus O and Sn is less than 2, indicating the presence of abundant oxygen vacancies.

**Figure 4:**
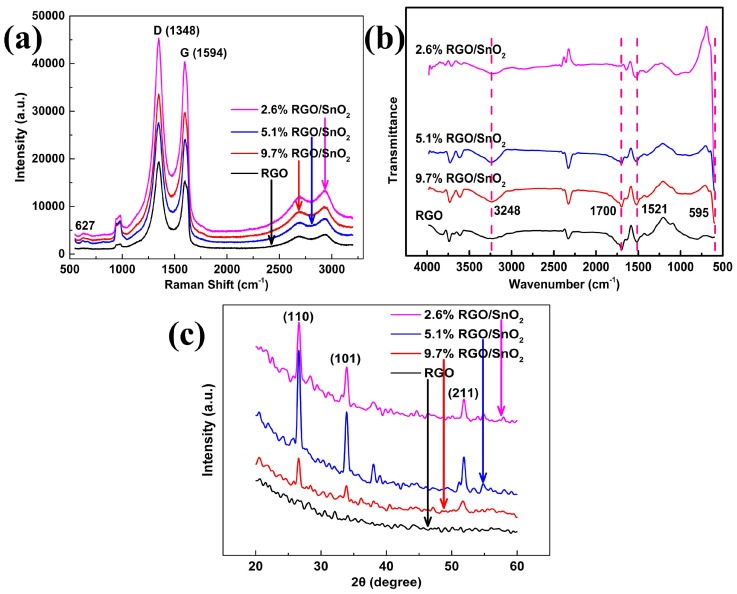
(**a**) Raman spectra. (**b**) FTIR spectra. (**c**) XRD patterns.

Raman spectra are commonly used to characterize graphene-based materials. The Raman spectra of the RGO film and the RGO/SnO${}_{2}$ composite films in Figure 4a show two main peaks at 1348 cm${}^{-1}$ and 1594 cm${}^{-1}$, which correspond to the D and G bands of RGO respectively. The D band is related to the structural defects and partially-disordered structures in the material, while the G band assigns to the graphitic hexagon-pinch mode [8,9,10]. The weak peaks located at around 627 cm${}^{-1}$ are attributed to SnO${}_{2}$[12]. Both characteristic peaks of RGO and SnO${}_{2}$ can be seen in the Raman spectra of the composite films, further evidence for the formation of RGO/SnO${}_{2}$ nanocomposites.

Raman $I_{D}/I_{G}$ ratio (where $I_{D}$ and $I_{G}$ are the D-band and G-band integral area values) are often used to evaluate the disorder and defects of RGO based materials [9,10]. $I_{D}/I_{G}$ of pure RGO, 9.7%RGO/SnO${}_{2}$, 5.1%RGO/SnO${}_{2}$ and 2.6%RGO/SnO${}_{2}$ films are calculated to be 1.77544, 1.58528, 1.67082 and 1.58917, respectively. Obviously, there are abundant surface defects and functional groups in RGO film. The lower $I_{D}/I_{G}$ ratios of the RGO/SnO${}_{2}$ nanocomposite films indicate that the introduction of SnO${}_{2}$ particles results in a further reduction of RGO. Compared with other composite films, 5.1%RGO/SnO${}_{2}$ film has more surface defects.

The FTIR spectra of RGO and RGO/SnO${}_{2}$ nanocomposite films are shown in Figure 4b. The typical peaks at 595 cm${}^{-1}$ for RGO/SnO${}_{2}$ nanocomposite films reveal the presence of the vibration mode of Sn-O from SnO${}_{2}$[5,13,14], which is more obvious with increasing the amount of SnO${}_{2}$. The broad IR absorption bands at 3248 cm${}^{-1}$ are attributed to O-H stretching vibration of adsorbed water (H${}_{2}$O) molecules [5,13], and its adsorption is related to surface defects. Among the composite films, the IR absorption band of 5.1%RGO/SnO${}_{2}$ film is the strongest, indicating the more surface defects, which is consistent with the results of Raman spectra. The other IR peaks can mainly be related to the RGO, in which the appeared peaks between 1521∼1700 cm${}^{-1}$ are due to C=C vibrations from aromatic carbon [13,14], which confirm the existence of RGO in the RGO/SnO${}_{2}$ nanocomposite films. The XRD patterns of RGO film and RGO/SnO${}_{2}$ nanocomposite films are presented in Figure 4c. The XRD patterns of RGO/SnO${}_{2}$ nanocomposite films show three main characteristic peaks corresponding to (110), (101) and (211), confirming the existence of SnO${}_{2}$ nanocrystals with tetragonal rutile structure (JCPDS card no. 41-1445). No obvious XRD peaks are detected for pure RGO film. As the amount of SnO${}_{2}$ nanopowders increases from 20 to 80 mg (the weight percentages of RGO decreases from 9.7% to 2.6%), the intensity of the characteristic peaks enhances first and then weakens, and get its maximum at 40 mg (5.1%RGO/SnO${}_{2}$). The enhanced peak intensity is due to the increased SnO${}_{2}$ nanoparticles in the composite film, while the more uniform distribution of the nanoparticles is responsible for the weakened peak intensity.

### 3.2. Influence of SnO${}_{2}$ Nanopowder Amount

**Figure 5:**
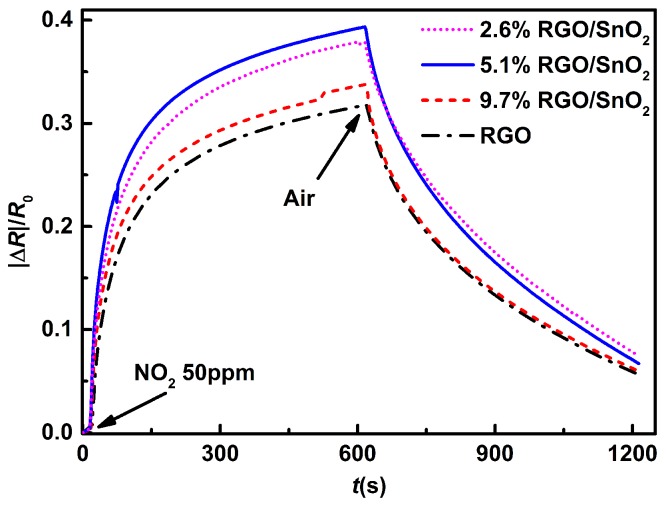
Responses of the sensors to 50 ppm NO${}_{2}$ at room temperature.

**Figure 6:**
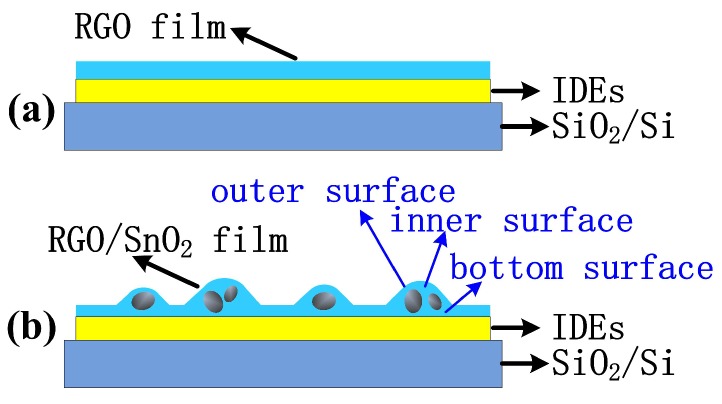
Schematic diagrams of (**a**) RGO film (**b**) RGO/SnO${}_{2}$ composite film.

Figure 5 displays the response behaviors of the sensors based on RGO, 9.7%RGO/SnO${}_{2}$, 5.1%RGO/SnO${}_{2}$ and 2.6%RGO/SnO${}_{2}$ films upon exposure to 50 ppm NO${}_{2}$ at room temperature (298.15 K). It can be seen that the combination of SnO${}_{2}$ nanoparticles improves the response of RGO sensor. However, the excessive amounts of SnO${}_{2}$ deteriorates the response of the sensor based on RGO/SnO${}_{2}$ composite film. The enhanced NO${}_{2}$ sensing properties can be attributed to the increased number of active sites for NO${}_{2}$ adsorption caused by the increased surface roughness of the sensing film and the surface defects. The corresponding mechanism is shown in Figure 6. The RGO sheets surrounding the SnO${}_{2}$ particles have at least three contact surfaces, that is, the outer surface and inner surface belonging to the skin layer and the bottom surface that contact with the IDEs. Thus, the composite film possesses more active adsorption sites for NO${}_{2}$ molecules than the pure RGO film. This results in the increased holes in the RGO/SnO${}_{2}$ composite film (RGO acting as the conducting layer), owning to the fact that more electrons transfer to NO${}_{2}$ molecules. Correspondingly, the resistance change caused by the NO${}_{2}$ adsorption is more remarkable, and the response increases.

### 3.3. Influence of Annealing

As a matter of fact, more active adsorption sites means a more difficult desorption of NO${}_{2}$ molecules adsorbed on the RGO/SnO${}_{2}$ composite film, which leads to a worse baseline drift [15].

**Figure 7:**
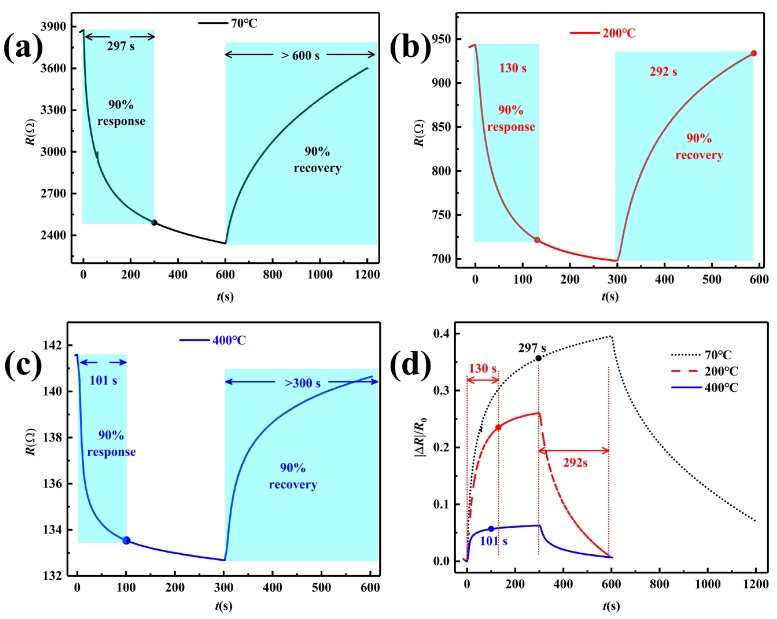
Response and recovery curves of sensors toward 50 ppm NO${}_{2}$. (**a**–**c**) Real-time resistance curves of sensors annealed at 70, 200, 400 ${}^{\circ }$C, (**d**) real-time response curves.

Therefore, the 5.1%RGO/SnO${}_{2}$ sensors were annealed in N${}_{2}$ ambient for an hour at 70, 200, 400 ${}^{\circ }$C, respectively. The resistance and response change curves are shown in Figure 7. As the heat treatment temperature increases, it can be seen from Figure 7a–c that the sensor resistance decreases, which might be due to the further reduction of RGO. Therefore, the oxygen-containing groups and surface defects in the composite films decrease, increasing conductivity, whereas reducing the interaction between the NO${}_{2}$ molecules and the sensing film. Although the adsorbed NO${}_{2}$ molecules are easier to be desorbed and the response time decreases from 297 s to 101 s (Figure 7d and Table 1), the response of the sensor annealed at 400 ${}^{\circ }$C to 50 ppm NO${}_{2}$ decreases significantly from 0.396 to 0.0629, which is only 15.9% of the original and becomes insignificant. The maximum response of the sensor annealed at 200 ${}^{\circ }$C decreases too, but it reaches 0.26 and retains 65.7% of the response of the sensor annealed at 70 ${}^{\circ }$C. More importantly, the sensor annealed at 200 ${}^{\circ }$C can recover fully (with recovery time of 292 s), which is beneficial to reduce the baseline drift and enhance sensing stability, so it is the best of the three annealed sensors.

### 3.4. NO${}_{2}$ Sensing Performance

**Figure 8:**
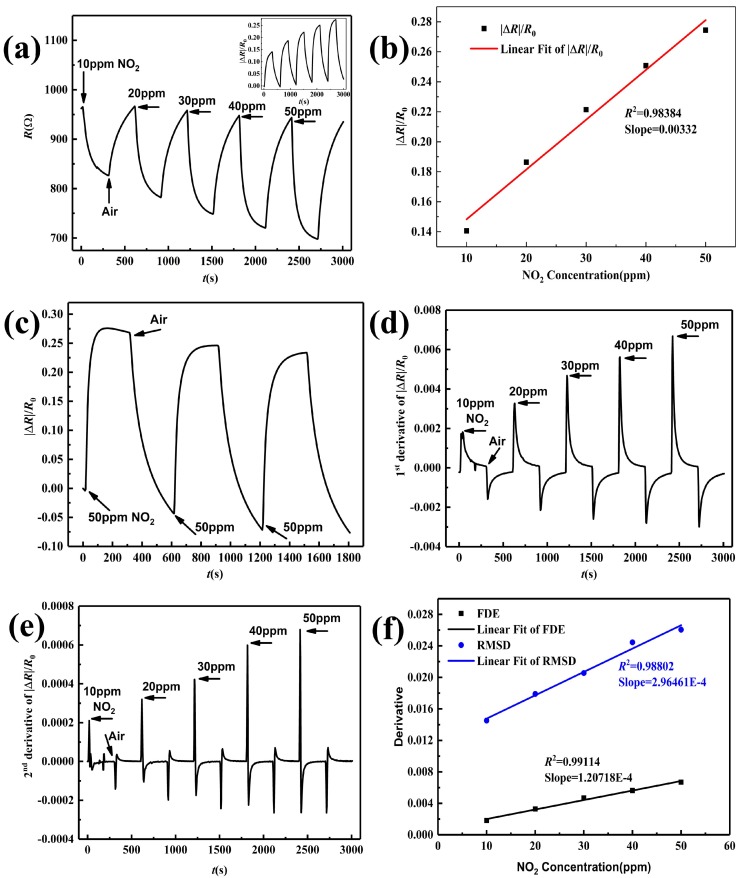
Sensing performance of 5.1%RGO/SnO${}_{2}$ sensor (**a**) Real-time resistance change and response, (**b**) Sensitive, (**c**) Repeatability, (**d**) first derivative of response, (**e**) second derivative of response. (**f**) linear fit of FDE and RMSD.

Figure 8a displays the real-time resistance change of 5.1%RGO/SnO${}_{2}$ sensor annealed at 200 ${}^{\circ }$C upon exposure to 10–50 ppm NO${}_{2}$, and the response curve is given in the inset. The resistance decreases quickly and reach a saturated state slowly as NO${}_{2}$ is injected, and then increases to the baseline as NO${}_{2}$ is purged by air. The sensor response increases with increasing NO${}_{2}$ concentration. The linear relationship between the response and gas concentration is significant ($R^{2}=0.98384$), which can be seen from Figure 8b. To evaluate repeatability, the response curve of the sensor toward 50 ppm NO${}_{2}$ during three cycles is given in Figure 8c, from which the response and recovery can basically maintain stability. This indicates that the as-fabricated sensor can detect NO${}_{2}$ at room temperature with a small baseline drift. Along with our previous work [16], the First Derivative Extrema (FDE) and the square Root of Maxima of Second Derivative (RMSD) are both proportional to the gas concentration and not affected by the baseline drift, so the velocity and acceleration of the response is calculated, the corresponding curves are demonstrated in Figure 8d,e. The extrema of the 1st and 2nd derivative increase with the enhancement of NO${}_{2}$ concentration, and they can be used to calibrate gas concentrations. The FDE and RMSD are extracted, and their linear fitting in Figure 8f exhibits better linearity with *R*${}^{2}$ 0.99114 and 0.98802, respectively.

### 3.5. Selectivity

**Figure 9:**
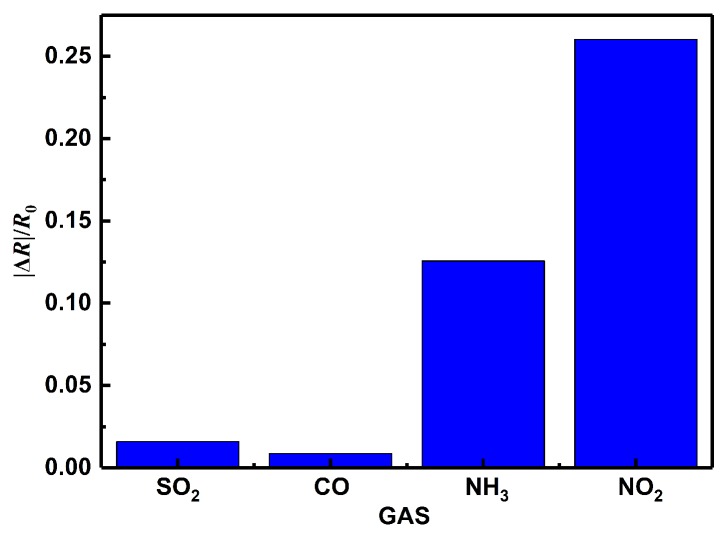
Selectivity of 5.1%RGO/SnO${}_{2}$ sensor to 50 ppm SO${}_{2}$, CO, NH${}_{3}$, NO${}_{2}$.

Figure 9 shows the sensing response of 5.1%RGO/SnO${}_{2}$ sensor annealed at 200 ${}^{\circ }$C toward 50 ppm of various interfering gases including SO${}_{2}$, CO, NH${}_{3}$ and NO${}_{2}$ at room-temperature. The response towards NO${}_{2}$ is 0.26034, much higher than that towards other gases.

Compared with the previously reported sensors based on RGO/ SnO${}_{2}$ hybrids [10,15,17] as shown in Table 2, the 5.1%RGO/SnO${}_{2}$ sensor annealed at 200 ${}^{\circ }$C in this work shows a greater potential in sensing NO${}_{2}$ at room temperature. Its good linearity, large detection range, and smaller baseline drift are beneficial to the relatively stable response and recovery. The sensors [10,17] have good responsiveness, response time and recovery time in a small measurement range, however, the linearity of the sensor in this paper is better when the concentration of NO${}_{2}$ exceeds 20 ppm.

## 4. Conclusions

A room temperature chemoresistive NO${}_{2}$ sensor based on an RGO/SnO${}_{2}$ nanocomposite film was fabricated via an air-brush spray deposition process. Therein, the aqueous suspensions of RGO/SnO${}_{2}$ composites acted as the spraying raw materials, which were prepared by mixing the diluted RGO dispersion and the SnO${}_{2}$ nanopowder. Compared with the pure RGO film, the RGO/SnO${}_{2}$ composite films provide more adsorption sites for NO${}_{2}$ molecules because of their rough surface, and thus have better NO${}_{2}$ sensing properties. In addition, the effects of the SnO${}_{2}$ content and heat treatment temperature on the NO${}_{2}$ sensing properties of the RGO/SnO${}_{2}$ nanocomposite film were investigated. The optimum composite and heat treatment temperature are 5.1%RGO/SnO${}_{2}$ and 200 ${}^{\circ }$C, respectively. In this case, a small baseline drift, wide detection range, high stability and excellent selectivity are achieved for the as-fabricated NO${}_{2}$ sensors. Finally, we demonstrate the superiority of the FDE and RMSD of the response curves in detecting NO${}_{2}$. 

## Figures and Tables

**Table 1:** Response and recovery time of sensors with different annealing temperatures to 50 ppm NO${}_{2}$.

Annealing Temperature (${}^{\circ }$C)	Response	Response Time (s)	Recovery Time (s)
70	0.396	297	>600
200	0.260	130	292
400	0.0629	101	>300

**Table 2:** Comparison of NO${}_{2}$ sensing performances of our as-prepared sensor with other published RGO/SnO${}_{2}$ sensors

Materials	Concentra- Tion (ppm)	Opterating Temperature (${}^{\circ }$C)	Response	Response /Recovery Times (s/s)	Range of Linearity (ppm)	Baseline Drift	Reference
RGO-CNTs-SnO${}_{2}$	5	RT	0.605	8/77	1–10	-	[17]
RGO-SnO${}_{2}$	5	RT	about 0.222	288/619	-	-	[17]
SnO${}_{2}$/N-RGO	5	RT	0.275	45/168	1–20	not obvious	[10]
SnO${}_{2}$/RGO	5	RT	about 0.241	415/740	-	-	[10]
RGO/SnO${}_{2}$ 10 mg/mL	25	RT	about 0.22	-/-	10–25	obvious	[15]
5.1%RGO/SnO${}_{2}$	50	RT	0.260	130/292	10–50	not obvious	this paper
